# Drafting a blueprint for the design of a rare disease ecosystem in Slovenia: Identifying salient opportunities and outlining policy recommendations

**DOI:** 10.7189/jogh.11.03064

**Published:** 2021-08-31

**Authors:** Dalibor Stanimirovic, Eva Murko, Tadej Battelino, Urh Groselj, Mojca Zerjav Tansek

**Affiliations:** 1 National Institute of Public Health, Ljubljana, Slovenia; 2 University Children’s Hospital Ljubljana, Department of Endocrinology, Diabetes and Metabolism, Ljubljana, Slovenia; 3 University of Ljubljana, Faculty of Medicine, Ljubljana, Slovenia

In line with some indicative estimations, there are approximately 150 000 patients with rare diseases (RDs) in Slovenia [1]. Notwithstanding the scarcity of dependable epidemiological data, the outlined figures signify that this field is of serious concern for the Slovenian health care system [2], which remains considerably incapacitated in terms of health care resources, operating efficiency, and long waiting periods for some health care services. Besides the overall lack of resources and know-how in the field, RDs frequently have complicated features, which additionally intensify their burden to the Slovenian health care system. It can be noted that the general problems faced by health care systems in managing RDs extend to various complex factors [3]. The inclusion of these factors in a new coherent and functional structure requires innovative changes to the existing arrangement in the field of RDs. Such new and innovative constellations in individual areas, which include human stakeholders and all related tangible and intangible elements, and their interrelationships, are increasingly being called *ecosystems* [4]. The ideal ecosystem in the field of RDs is thus considered to be a functional environment that incorporates all stakeholders, mechanisms, and rules for coordinated and comprehensive patient treatment.

A large part of these components already exists in Slovenia and operates well (also on an international scale), but at the same time these components are not integrated in an organized ecosystem and some of them are rather deficient and inactive, and lack opportunities for more effective collaboration and performance. The entire field of RDs is thus confronted with developmental issues, high operating costs, and the inability to exploit the existing health care system capacities and institutional synergies to meet the growing and increasingly diverse needs of RD patients. In order to develop an ecosystem in the field of RDs in the true sense of the word, it is necessary to set up an adequate strategic framework, integrate all these components, manage their operation, and mitigate the problems associated with the coordination of stakeholders and the lack of resources. The required integration of the ecosystem components can only be realized by appropriately leveraging information and communication technology (ICT) solutions, which should become the main facilitator enabling the establishment of a collaborative, functional, and process-oriented RD ecosystem. Drawing on experience from the field and the detected starting points, the authors’ initiative proposes a draft of a blueprint for a national RD ecosystem in Slovenia. Proceeding from the assumption that the ecosystem will indeed be instituted in the foreseeable future, the authors have identified salient opportunities offered by the establishment of the RD ecosystem, and outlined policy recommendations for the realization thereof.

## DETECTING STARTING POINTS AND DRAFTING A BLUEPRINT FOR THE DESIGN OF A RARE DISEASE ECOSYSTEM IN SLOVENIA

In order to successfully establish an RD ecosystem, certain elementary preconditions must be met. Strategic orientations in this sense represent a *conditio sine qua non* of the successful institution of an RD ecosystem. The lack of both a long-term strategic framework and current operational policies pose serious problems in the field of RDs, which are reflected in all connected areas and have a very negative impact on all related factors in the existing system in the field of RDs. The only relevant document in the field is the “Work plan in the field of rare diseases in the Republic of Slovenia” [1], which expired last year. Regrettably, up-to-date strategic documents and action plans for the future have yet to be prepared. Intermittent interest and a lack of commitment among health care policymakers in the field generally result in insufficient funding and the poor engagement of stakeholders. Such conditions typically undermine all efforts to establish an ecosystem.

The number of institutions dealing with RDs in Slovenia is relatively small. Patients with RDs are in most cases treated and rehabilitated at five institutions. The small size of Slovenia and the unified public health system with one financier should represent an advantage in treating RD patients. However, due to the evident management crisis in the Slovenian health care system and inertia from the previous years, there are considerable opportunities for organizational improvements, especially with regard to the coding process, better communication, the utilization of institutional capacities and resources, the coordination and streamlining of work processes, and cooperation in planning the continuous treatment of RD patients [5].

**Figure:**
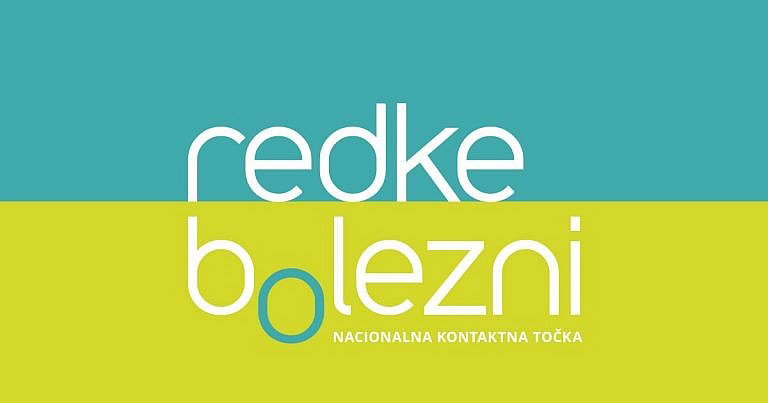
Photo: Logo of the National Contact Point for rare diseases in Slovenia (https://www.redkebolezni.si/, used with permission.

Health care professionals working in the field of RDs do not have any specialized ICT solutions for local outpatient work or central ICT solutions to collect, analyse, and compile data on RDs at the national level – such as a national RD registry [6]. A national RD registry is envisaged as one of the essential components of the RD ecosystem, providing a means for monitoring and managing RDs in Slovenia and consequently improving the treatment of patients with RDs. An RD registry represents a central tool for the impartial collection and processing of data on RDs, clinical research, and population studies, and may significantly improve the access of all stakeholders involved in the RD ecosystem to relevant data. Therefore, the development of interoperable ICT platforms and RD registries is one of the EU’s priorities in the management of RDs, as evidenced by different health care resolutions, strategic documents, and EU-funded projects [7,8]. On the other hand, the National Contact Point (NCP) for RDs established in 2016, should play a very important role in providing comprehensive support to patients in the field of RDs. The goal of the NCP is to create a network of stakeholders and to provide patients, their families, and experts access to high-quality information on the treatment of RDs in Slovenia. The empowerment of patients should increase their capacity to express needs and concerns [9], but it should also encourage them to get involved in the processes that help them gain control over their own lives.

**Figure 1:**
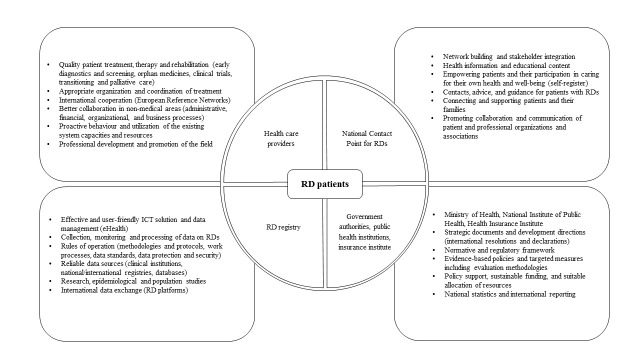
The blueprint for the design of an RD ecosystem in Slovenia.

Arising from the starting points and practice in the field as well as the primary needs of the stakeholders, we drafted a blueprint of the preferred RD ecosystem in Slovenia (**Figure 1**). The planned RD ecosystem in Slovenia should strive to broadly involve all relevant stakeholders, build up their capacities, functionalities, and mutual relationships, and support them with operative ICT tools and mechanisms, as illustrated in **Figure 1****.**

## IDENTIFYING SALIENT OPPORTUNITIES AND OUTLINING POLICY RECOMMENDATIONS

Patients with RDs typically face late diagnosis, long-lasting and expensive treatment (if medication is even available), and social consequences [10]. For individuals affected by these largely unpreventable diseases, a comprehensive public health care approach, including the establishment of an operative RD ecosystem, would entail an important opportunity for more coherent and effective medical treatment [5]. Raising awareness of RDs and collaborative efforts in the field could significantly contribute to ensuring a more equitable allocation of resources, which is still extremely inadequate regarding the scope of the problem and comparison with some other diseases deemed to be public health priorities [2].

The Slovenian health care system operates relatively well in the field of RDs, given the available resources and other systemic circumstances that have already been mentioned. Nevertheless, there are considerable opportunities for progress that will need to be addressed as comprehensively as possible. Forthcoming policies, actions, and practices will need to deal with these issues, since they could have a decisive impact on the establishment of an RD ecosystem in Slovenia. This implies that future government documents, measures, and clinical operation may have a critical effect, and either promote or significantly inhibit the desired development in the field of RDs. In view of this, there is compelling evidence from other social subsystems and industries that the establishment of an RD ecosystem represents one of the foundations for the systemic regulation of RDs in the country [4]. Similar initiatives towards the development of ecosystems can be found in education, culture, business, entrepreneurship, innovation, technology, etc. [11-16]. Every component outlined in the blueprint has a specific role in the proposed ecosystem constellation and should provide a significant contribution to the overall functioning of the RD ecosystem, and offer benefits, be it for patients, health care professionals, or the health care system in general.

Future policy initiatives should advocate ecosystem-oriented transformations and approaches in the field of RDs. A well-organized RD ecosystem with its operative constituent components could substantially contribute to more comprehensive monitoring of RDs, improved, and better coordinated patient treatments, reduced inequality, and more effective two-way communication between RD patients and other stakeholders. In addition, focusing on the strategic, financial, and development aspects, an ICT-based RD ecosystem could convey significant benefits to all health care decision-makers by enhancing evidence-informed policymaking, providing a convenient platform for the estimation and allocation of the required resources, and facilitating institutional reorganization, and technological innovations, including predictive analytics and artificial intelligence. Nevertheless, it is evident that the formation of an inclusive RD ecosystem necessitates in-depth reform of the existing arrangements, including the commitment of stakeholders, sustained by focused policy measures and appropriate programmes.

The prospective policies for the integration of the outlined components and the establishment of the RD ecosystem will have to be grounded in a feasible strategy that accurately delineates the roles, assignments, and competences of each constituent component. Due to the complexity and significance of the components involved, the RD ecosystem will inevitably call for the efficient collaboration of the stakeholders, well-designed ICT tools and data management platforms, and an unwavering focus on the success of the treatment of patients and their general well-being. All of these efforts will have to be backed by substantial funding and the support of the highest health care authorities.

Despite the outlined challenges, recent developments concerning RDs in Slovenia illustrate that the establishment of an RD ecosystem would represent a significant advance in the field and bring tangible benefits for individual patients, on one hand, and enhance the effectiveness of public health policies by facilitating better utilization of health care resources, on the other.
